# Circulating miRNAs as Tumor Biomarkers: A Preface to the Special Issue

**DOI:** 10.3390/cancers12123836

**Published:** 2020-12-18

**Authors:** Athina N. Markou

**Affiliations:** Analysis of Circulating Tumor Cells, Laboratory of Analytical Chemistry, Department of Chemistry, University of Athens, 15771 Athens, Greece; atmarkou@chem.uoa.gr; Tel.: +30-2107274319

Nowadays, therapeutic strategies in cancer are subsequently defined according to the molecular profile of the tissue. Recent studies have established that targeted therapies may fail to cure the disease because of several biological and technological challenges. In light of these limitations on the use of tissue biopsies, new ways to observe tumor genetics and tumor dynamics have evolved. Liquid biopsy has been presented as a new diagnostic and therapeutic concept predicated on the analysis of: circulating cell-free DNA (cfDNA), circulating tumor cells (CTCs), cell-free RNAs and extracellular vesicles.

Circulating free RNAs (cfRNAs), either as a free molecule alone or in complexes with proteins and lipids, are present in various types of body fluids, such as blood, serum, and plasma [1]. There are several types of cfRNAs that could be distinguished based on their length and function: microRNA (miRNA), PIWI-interacting RNA (piRNA), tRNA-derived RNA fragments (tRFs), circular RNA (circRNA), long non-coding RNA (lncRNA), and messenger RNA (mRNA) [2].

miRNAs are endogenous non-coding small molecules (19–22 nt) that are involved in posttranscriptional regulation, and participate in various physiological and pathological processes by inhibition of translation, or by mRNA cleavage. Due to their protection from endogenous RNAs, miRNAs are the most stable and therefore the most well-studied cfRNAs molecules. miRNAs were secreted into the circulation either through being released from apoptotic and necrotic cells, or through active release from tumor cells [3,4].

In the last few years, circulating miRNAs have been studied (a) as promising non-invasive cancer biomarkers, even in early diagnosis [5], (b) as diagnosis biomarkers to distinguish different subtypes of cancer [6], (c) as prediction markers for the sensitivity of tumors to clinical treatment [7], (d) as prognostic biomarkers [8], and, finally, (e) as therapeutic targets. It is now accepted that these small molecules are involved in various cancer-associated biological processes, such as proliferation, differentiation, apoptosis, metabolism, invasion, metastasis, and drug resistance.

Moreover, it is crucial to note that the expression levels of circulating miRNAs were found to be significantly influenced by several pre-analytical and analytical factors that explain the poor overlap of results from different studies [9,10]. Validation and standardization of all preanalytical and analytical procedures is an imperative issue to successfully translate miRNA signatures in clinically meaningful tests.

Although the most common methods for circulating miRNAs detection are RNA sequencing (RNA-Seq), microarrays, and reverse transcription PCRs (RT-qPCR), there is a need for more reliable miRNA detection platforms that could be used routinely in laboratories, and improved the sensitivity and selectivity of miRNA detection [11]. The most widely used is relative quantification RT-PCR but this method requires a suitable internal control for the normalization of results, for which there are many conflicting views.

This Special issue will highlight the clinical importance of circulating miRNAs as biomarkers, the effects of various pre-analytical and analytical parameters on their measurements, as well as technological advances that will further improve our knowledge in the field of early diagnosis and the detection of minimal residual disease.
